# Editorial: Predictive tools in pheochromocytoma and paraganglioma

**DOI:** 10.3389/fendo.2023.1227543

**Published:** 2023-06-13

**Authors:** Filippo Ceccato, Ricardo Correa, Masha Livhits, Henrik Falhammar

**Affiliations:** ^1^ Department of Medicine DIMED, University of Padua, Padua, Italy; ^2^ Endocrine Disease Unit, University-Hospital of Padua, Padua, Italy; ^3^ Endocrinology, Diabetes and Metabolism, The University of Arizona College of Medicine Phoenix, Phoenix, AZ, United States; ^4^ Department of General Surgery, UCLA David Geffen School of Medicine, Los Angeles, CA, United States; ^5^ Department of Endocrinology, Karolinska University Hospital, Stockholm, Sweden; ^6^ Department of Molecular Medicine and Surgery, Karolinska Institutet, Stockholm, Sweden

**Keywords:** pheochromocytoma, paraganglioma, predictive model, surgery, endocrine neoplasms

Pheochromocytoma (PCC) and Paraganglioma (PGL) are rare endocrine neoplasms, composed of chromaffin cells, often secreting catecholamines, characterized by particular clinical manifestations and often with a benign outcome after surgery (1, 2). PCCs and PGLs are grouped together in the same syndrome, known as PPGL in combination.

Excessive catecholamine secretion should be considered in patients with secondary hypertension, especially in those with common signs and symptoms of PPGL: spells with hypertensive crisis, headaches, sweating and palpitations (3). The most sensitive screening test is the measurement of plasma or urinary fractionated metanephrines (4), considering that false positive results are reported with some common drugs (antidepressant, beta blockers and glucocorticoids) or foods (that contain tyramine such as banana, cheese, chocolate, red wine, coffee, and soya sauce) (5). Blood sampling conditions can alter results, with reduced diagnostic accuracy (in particular falsely elevated plasma normetanephrine concentrations) observed in low temperature, a semi-recumbent position, and direct venipuncture (6).

Adrenal incidentalomas, which affect up to 10% of adults older than 70 years (7, 8), are the most common presentation of PCCs (9, 10). Moreover, the prevalence of PPGL in outpatients with hypertension ranges between 0.2 and 0.6% (11). Ruling out PCCs is recommended in all adrenal incidentalomas according to the European Guidelines (12): sometimes PCCs can be detected by imaging before clinical signs/symptoms development, and absence of specific symptoms and hemodynamic features has been reported in half of patients with PCCs (13). The classic triad of symptoms (headaches, sweating, and palpitation) in combination with hypertension is only present in 28% of patients with suspected PCC (9).

After the endocrine and clinical diagnosis, cross-sectional imaging should be offered to patients with suspected PPGL. On computed tomography (CT) scan, PCCs have unenhanced Hounsfield Unit (HU) >10 with avid arterial enhancement and delayed venous washout, although one-third of PCCs have imaging characteristics that over-lap with lipid-poor adenomas (unenhanced HU>10 with rapid contrast washout) (14). On magnetic resonance imaging (MRI), T2 signal hyper-intensity (the light-bulb sign) is common in PCC (15). In a collaborative European study with CT scan, only 2out of 376 patients with PCC presented a high lipid content (unenhanced HU <10), therefore it was concluded that biochemical testing for PCC is not routinely necessary for lipid-rich adrenal incidentalomas (14). According to the American Association of Endocrine Surgeons Guidelines, preoperative blockade with selective or nonselective alpha blockers should be used to prepare patients with PPGL for surgery (16).

However, in clinical practice several challenges must be faced in patients with PPGL. Diagnosis and management are challenging if presentation is an adrenal incidentaloma, in subclinical or “silent” cases without overt secreting neoplasms, in case of known genetic variants and inherited disease, metastatic disease, and other peculiar situations such as children, pregnancy and elderly (17). The historical rule of 10% (10% non-adrenal, 10% non-unilateral, 10% malignant, 10% paediatric, and 10% syndromic) is no longer valid, and in the era of personalized and patient-centric approach, predictive tools are needed to correctly identify the outcome of the disease.

Among predictive tools in cancer research, the big-data approach of texture analysis, termed radiomics, is an increasing field of interest. Radiomics uses image-based texture analysis from conventional imaging to provide quantitative parameters that may be useful to measure the heterogeneity of tumours (18), and has been proposed to differentiate between benign and malignant adrenal lesions (19). Texture analysis was studied in a small number of patients (17 with surgically confirmed PPGL) and some selected features were able to identify secreting or malignant tumours (20). Patients with germline pathogenic variants involved in multiple cellular processes (hypoxia, MAPK/ERK or WNT signaling) presented a particular clinical picture with head/neck or thoracic/abdominal PGLs, secreting or not, and the genotype was able to predict the clinical behaviour (21). The number of known involved genes and their variants continues to grow. In the last 10 years, a European Consortium of Researchers developed and evaluated an omics-based stratified health promotion program for patients with endocrine forms of hypertension. A Machine Learning pipeline was able to differentiate essential hypertension from endocrine forms of secondary hypertension, combining miRNAs, plasma methylated metabolites, plasma steroids, urinary steroid metabolites, and plasma small metabolites (22).

The current Research Topic, “*Predictive Tools in Pheochromocytoma and Paraganglioma*” helps to bridge the gap between the clinical presentation and the diagnosis as well as management of PPGL by highlighting recent research findings. An increased awareness, combined with predictive tools and use of conventional and innovative techniques, may enhance the diagnosis of PPGL.

The paper by Zhao et al. considered the predictive role of the baseline size of the primary tumour. They analysed 263 patients with PPGL, sorting the mass according to the threshold usually used to consider a suspected adrenal mass: 110 patients were in the “small tumour” group (<4cm), and 153 patients were in the “large tumour” group (>4cm). Prevalence of male sex, hypertension, diabetes, hypertensive crisis, and elevated catecholamines secretion was higher in patients with small versus large PPGLs.

The research group of Wang et al. investigated the catecholamine-induced cardiomyopathy in patients with PPGL. Catecholamine-induced cardiomyopathy is a severe cardiac complication, resulting in heart failure and fatal arrhythmias (23). They conducted a 1:3 matched study (52 patients with and 156 without cardiomyopathy) and reported that the patients with catecholamine-induced cardiomyopathy were younger, with more clinical symptoms and signs at diagnosis, showing higher systolic and diastolic blood pressure levels, heart rate, 24-h urine catecholamines excretion, larger tumour diameter (median was >4cm in both groups), and increased presence of genetic syndromes, especially VHL and MEN.

The management of surgery, the cornerstone of therapy, in patients with PPGL is a clinical challenge where intraoperative hemodynamic instability can increase the intra-, peri- and post-operative risk (17). Zhang et al. calculated a nomogram, built on clinical and radiological parameters (age >60 years, tumour size, BMI, laterality, Mayo Adhesive Probability score, and necrosis) that can be used to stratify the surgical risk of patients.

Liu et al. studied 183 patients with lipid-poor adenomas and 86 patients with subclinical PCC. Unenhanced and enhanced attenuation values were higher in PCC. They also developed a prediction model for histology based upon several radiological features (shape, homogeneity of the texture, necrosis, or cystic degeneration).

In summary, this Research Topic illustrates that an old disease such as PPGL that has been defined more than 100 years ago still requires new knowledge and innovative research.

## Author contributions

FC: draft writing: RC, HF and ML: review and editing. All authors contributed to the article and approved the submitted version.
